# Investigation of Matlab^®^ as Platform in Navigation and Control of an Automatic Guided Vehicle Utilising an Omnivision Sensor

**DOI:** 10.3390/s140915669

**Published:** 2014-08-25

**Authors:** Ben Kotze, Gerrit Jordaan

**Affiliations:** 1 Department Electrical, Electronic and Computer Engineering, Central University of Technology, Free State, Private Bag X20539, Bloemfontein 9300, South Africa; 2 Research Group in Evolvable Manufacturing Systems, Central University of Technology, Free State Bloemfontein 9300, South Africa; 3 Technology and Innovation, Central University of Technology, Free State, Private Bag X20539, Bloemfontein 9300, South Africa; E-Mail: gjordaan@cut.ac.za

**Keywords:** omnidirectional, image processing, area of interest, Prewitt edge detection, Kalman filter, colour routes, reconfigurable paths, Matlab^®^

## Abstract

Automatic Guided Vehicles (AGVs) are navigated utilising multiple types of sensors for detecting the environment. In this investigation such sensors are replaced and/or minimized by the use of a single omnidirectional camera picture stream. An area of interest is extracted, and by using image processing the vehicle is navigated on a set path. Reconfigurability is added to the route layout by signs incorporated in the navigation process. The result is the possible manipulation of a number of AGVs, each on its own designated colour-signed path. This route is reconfigurable by the operator with no programming alteration or intervention. A low resolution camera and a Matlab^®^ software development platform are utilised. The use of Matlab^®^ lends itself to speedy evaluation and implementation of image processing options on the AGV, but its functioning in such an environment needs to be assessed.

## 1. Introduction

AGV sensors like infrared and ultrasonics are being replaced by using vision, which produces more information for controlling the vehicle. The AGV utilises a single digital camera providing omnidirectional (360°) vision for navigation [1]. A reconfigurable solution for manufacturers could be the reprogramming of such a vehicle to use alternative routes and keeping the operators' programming input to a minimum, rather than implementing altering conveyor systems for transporting goods.

**Figure 1.**
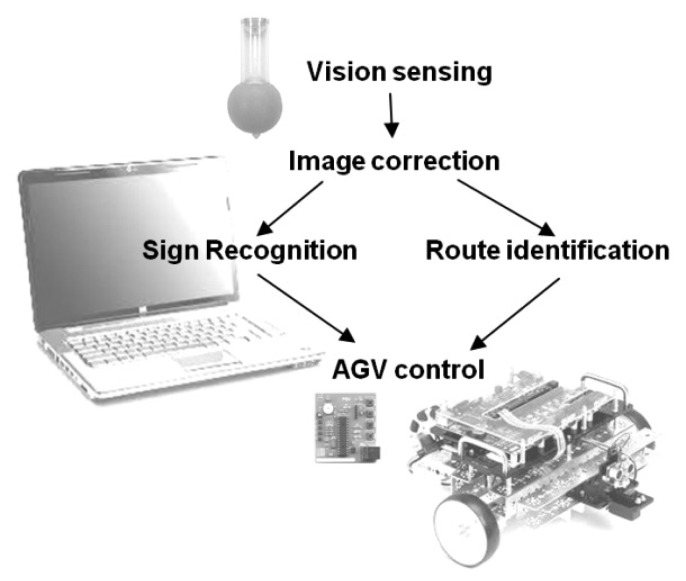
Illustrated layout of the complete system.

The project involved a vision sensor, AGV vision navigation control and the development of a reconfigurable approach to prove the feasibility of using a single software platform like Matlab^®^ for speedy evaluation and implementation of image processing options. An overview of the system is illustrated in Figure 1.

## 2. Vision Sensing

**Figure 2.**
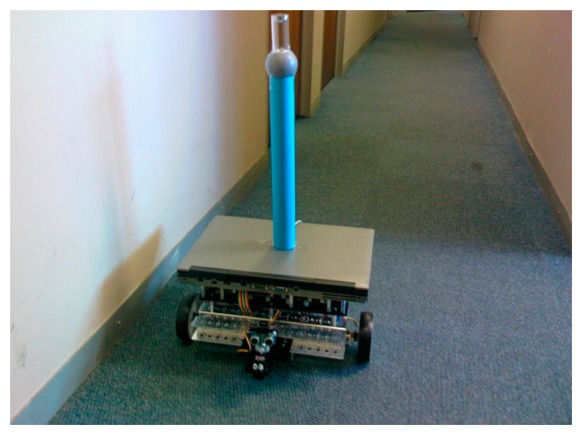
AGV platform using a laptop, NI robot platform and omnivision system.

As the surroundings were to be detected by vision, the setup used a webcam using an omni-mirror setup placed on top of a National Instruments (NI, Austin, TX, USA) robot platform is shown in Figure 2. All the processing and control was done by a laptop placed on the NI platform.

A PC was used for the development and initial simulations. The code was then transferred to a laptop for navigation trials. Table 1 lists the specifications of the PC and the laptop.

### 2.1. Omnidirectional Conversion for Vision Sensing

**Figure 3.**
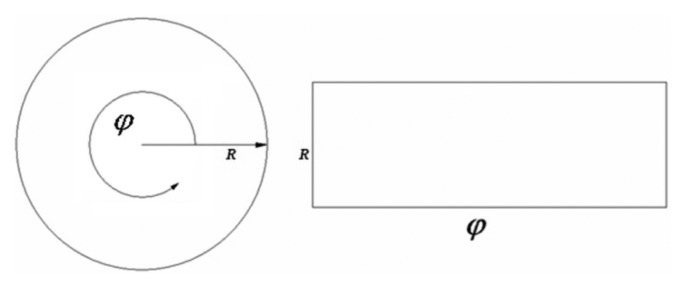
Graphical representation of a polar transform.

Scaramuzza's research proved that a polar transfer function can be implemented by creating a good panoramic image [2]. The polar transform implemented in Matlab^®^ applied Equation (1) shown below:

(1) $$\begin{array}{l} R=\text{radius}\\ \phi =\left(\varphi \times \frac{\text{resolution}}{180{}^{\circ}}\times \pi \right)\text{rad}\\ X_{\text{Pixel position}}=R\cos(\phi )+X_{\text{Centre offset}}\\ Y_{\text{Pixel position}}=R\sin(\phi )+Y_{\text{Centre offset}}\\ 0\leq R\leq \text{Maximum radius}\\ 0{}^{\circ}\leq \varphi \leq 360{}^{\circ} \end{array}$$

where *Maximum radius* represents the height of the frame to be converted and *φ* the resolution width of the panoramic view, as can be seen in Figure 3.

**Figure 4.**
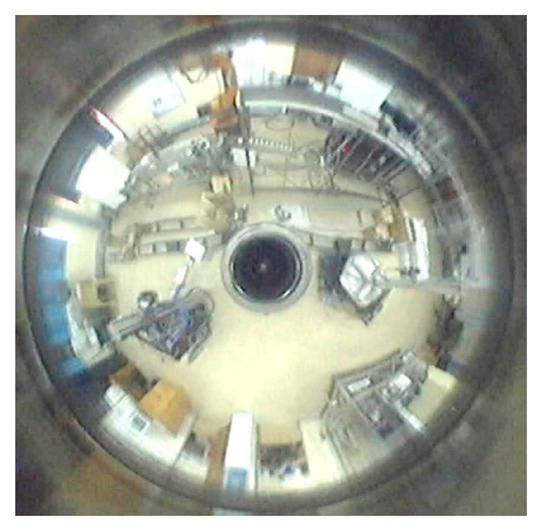
Environmental picture in circular (680 × 670 pixels) mirror image.

**Figure 5.**
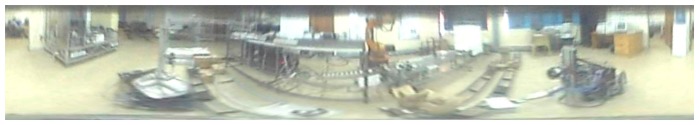
Transferred image of Figure 4, −90° corrected and mirror image effect corrected.

This polar transform was applied to Figure 4 using Matlab^®^ with the result shown in Figure 5 ([3], pp. 1835–1839).

**Figure 6.**
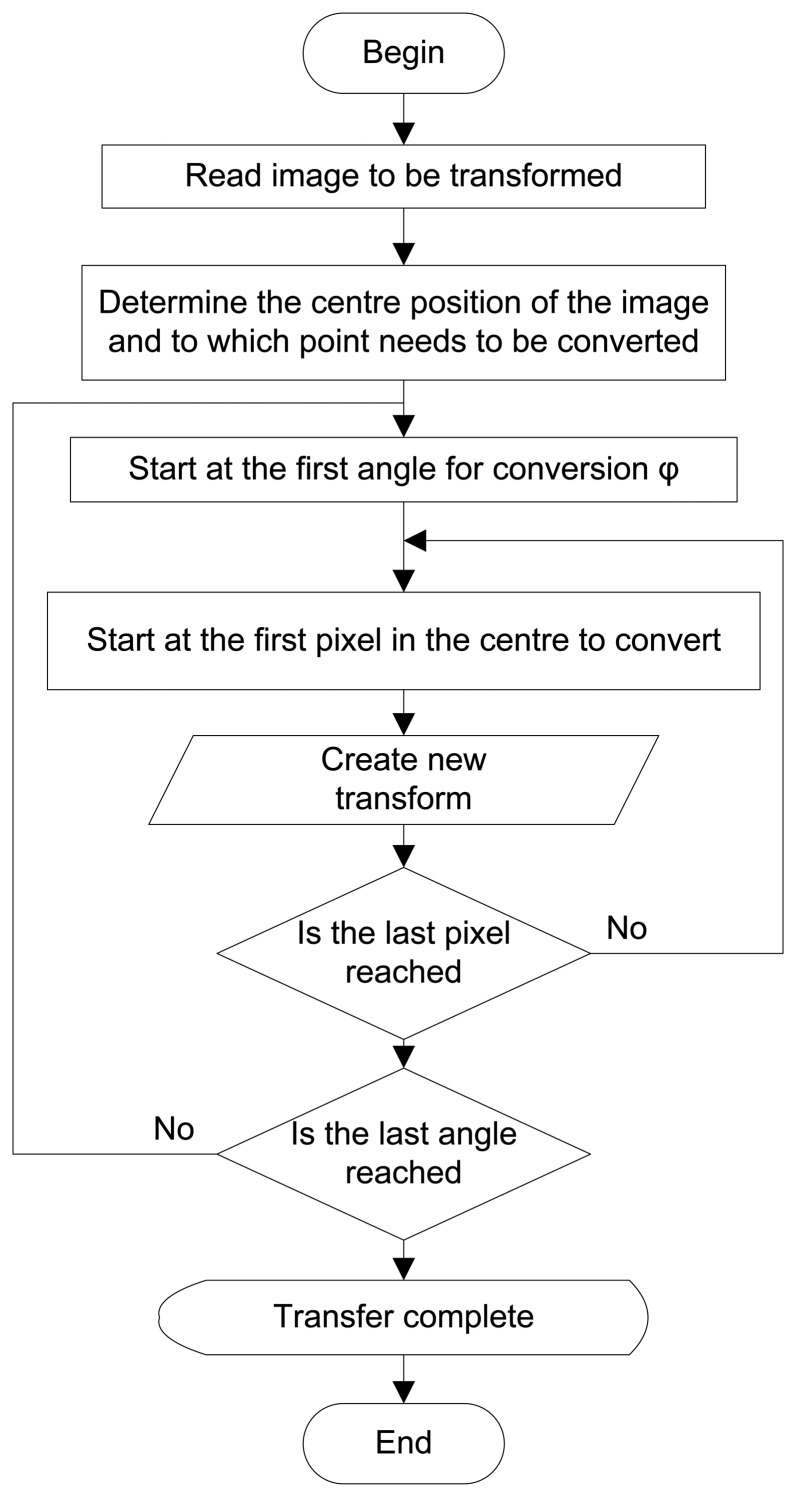
Flowchart for program—polar to Cartesian.

**Figure 7.**
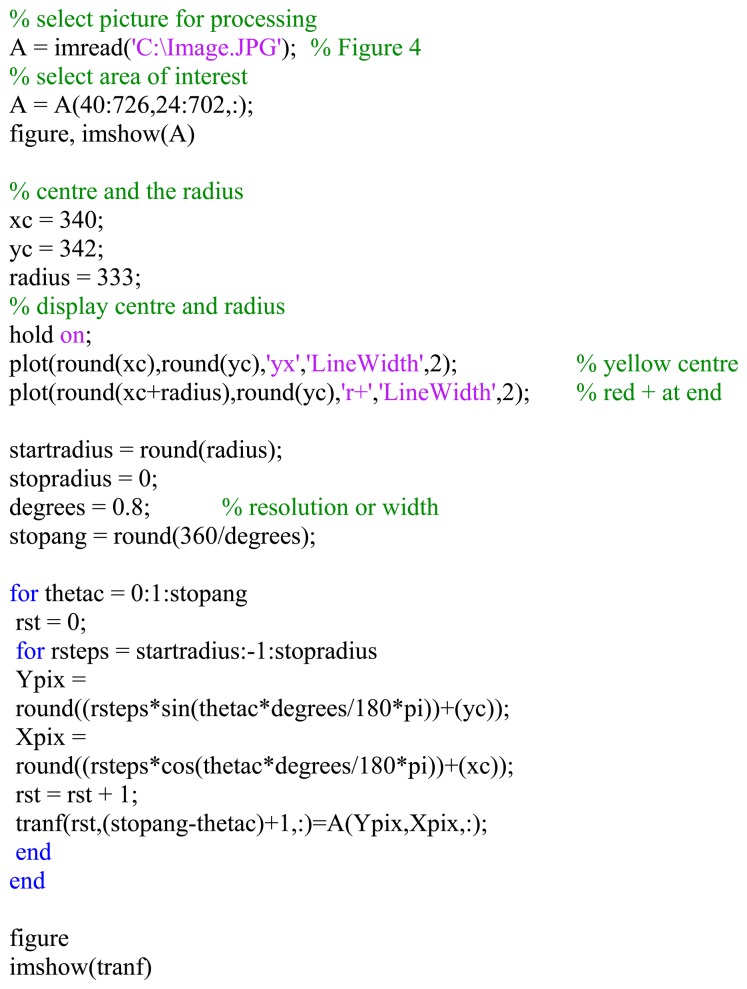
Matlab^®^ program extract—polar to Cartesian.

Figure 5 was generated with a Matlab^®^ function with a radial step resolution of 1° [4,5]. This function does the transform on pixel level and is very time consuming. It took almost 1 s for the image of 2.25 MB to be transformed with this function on an Intel^®^ Pentium^®^ 3.4 GHz CPU with 3.25 GB of RAM. Figure 6 shows the flowchart and Figure 7 an extract of the Matlab^®^ M-file providing the result.

### 2.2. Omnidirectional Sensing Software in MATLAB^®^

**Figure 8.**
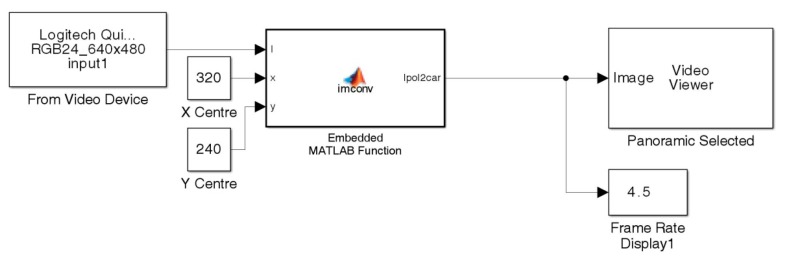
Simulink^®^ model for converting omnivision pictures to a panoramic picture stream.

The initial development was done on single pictures taken with the omnidirectional photographic setup. These were changed to a video streamed system. Simulink^®^ was incorporated for this purpose. Figure 8 shows a conversion model development for accessing the camera by utilising the *From Video Device* data block. The *Embedded MATLAB* function was written incorporating the conversion model seen in Figure 7.

#### Implementing the Concept of an Area of Interest

**Figure 9.**
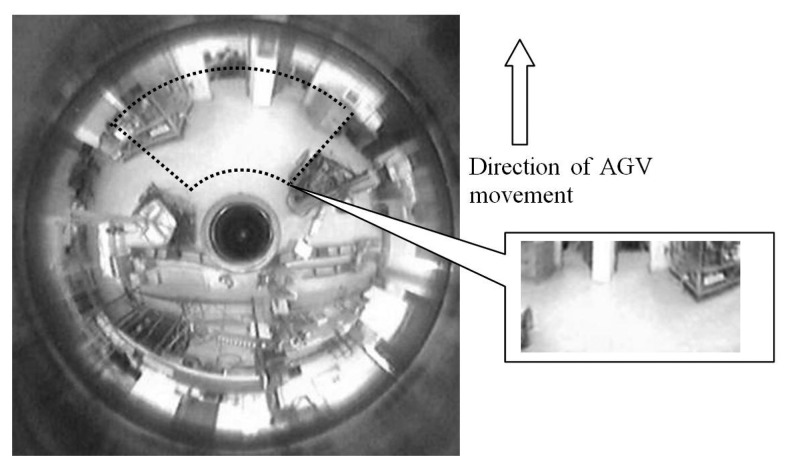
Illustration of capturing a frame, selecting an area of interest for conversion and final resolution for conversion.

With the results obtained in Section 2.1, it seemed imperative to reduce the computation time for acquisition, conversion and display. This could be done by selecting a smaller area of interest in the direction of movement of the AGV as illustrated in Figure 9.

### 2.3. Transferring the Omnivision Software from Computer to Laptop Platform

**Figure 10.**
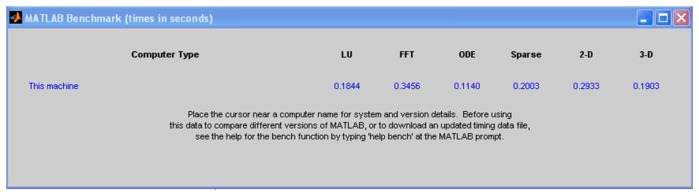
Matlab^®^ *bench* feature displayed as the PC's result, for the process speed in seconds.

**Figure 11.**
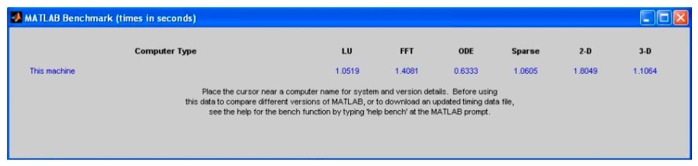
Matlab^®^ *bench* feature displayed as the laptop's result, for the process speed in seconds.

Matlab^®^ has a feature, called *bench*, to evaluate the processing strength of computers in different calculating areas. The result for the platforms used in Table 1 is shown in Figures 10 and 11.

Figures 10 and 11 clearly indicate that the particular PC used outperformed the laptop platform to which it was compared. The comparison data for other computer platforms are stored in a text file, “bench.dat”. Updated versions of this file are available from Matlab^®^ Central [6]. Keeping these results in mind, the results shown in Table 2 were obtained.

## 3. Navigation for the AGV Using Vision

In a reconfigurable environment it should preferably be possible to alter the route that the AGV needs to travel, depending upon ordering information (origin or pickup point) and delivery of parts (destination) [7]. This concept was assumed for evaluating the omnivision sensor implemented in Matlab^®^ for the navigation and control of an AGV.

### 3.1. Route Identification for Navigation

**Figure 12.**
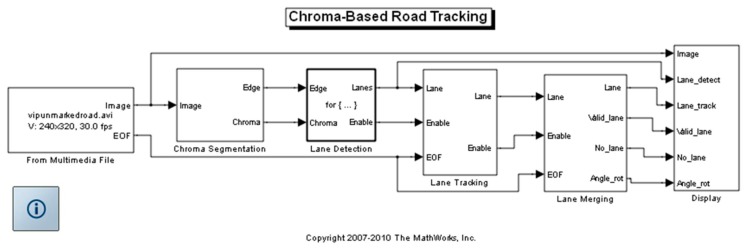
Matlab^®^ “*Chroma-Based Road Tracking*” demo.

Sotelo *et al.*'s work proved the use of lines on the side of a route or walkway, or alternatively a chroma route could be used for route navigation [8]. Matlab^®^'s “*Chroma-based Road Tracking*” was altered for this purpose. Figure 12 illustrates the Simulink^®^ model of the demo [9]. When running the demo a pre-recorded video was used as source to be processed for evaluating the road tracking concepts used. The model then used the chroma information of the frames to detect and track the road edges. The “*Chroma-based Road Tracking*” demo model illustrates the use of the *Colour Space Conversion* block, the application of *Hough Transform* block, and the advantage of the *Kalman filter* block to detect and track information using hue and saturation values of the frames from the video.

The demo model performs a search operation to define the left and right edges of a road by analysing video frames for a change in colour behaviour. The model then selects a line either because of an edge detected, or a line created by a change of chroma pixels, whichever has the greater precedence. The search is initiated from the bottom-centre of each frame and moves to both the upper-left and upper-right hand corners of each frame. From this model outputs were generated to navigate and control the AGV on a set route.

### 3.2. Laptop to Motor Speed Control Interface

There was a need to communicate the associated direction commands from the evaluation images of the camera system to the AGV platform. A PIC microcontroller board and software was developed in such a way that the direction control signal of the AGV was sent serially via the USB port of the laptop to the PIC board also using Matlab^®^'s serial communication data block.

## 4. Reconfigurable Approach Using Sign Recognition

**Figure 13.**
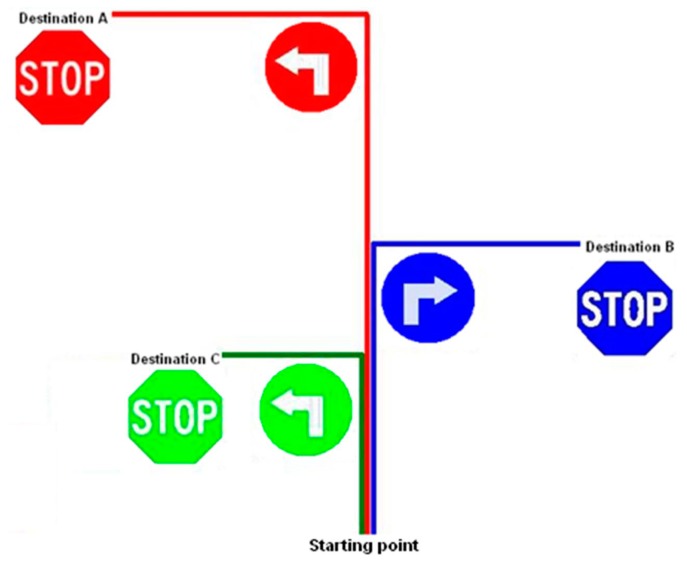
Incorporating signs for defining reconfigurable routes for multiple AGV's by using different colours.

The concept of sign detection in conjunction with route tracking is to provide the AGV controller with an indication as to which route is to be taken when encountering more than one option. This is accomplished by incorporating left- and right turn signs, with a stop sign at its destination. This gives the AGV a reconfigurable route set by the operator, without programming intervention or changes, by placing the required signs along a changeable route. Altering the colour which the AGV responds to, gave rise to alternative routes for different AGV's to follow, best illustrated by Figure 13.

**Figure 14.**
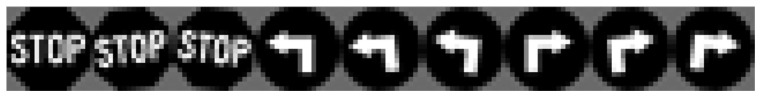
Three sign templates: stop, left and right; with three orientations each, 0° + 7.5° and −7.5°; generated for the recognition process.

Correlation was achieved by implementing templates of the signs after initial detection of the preset colour for the specific AGV. An example of the templates is shown in Figure 14.

### 4.1. Displaying the Recognition Results

**Figure 15.**
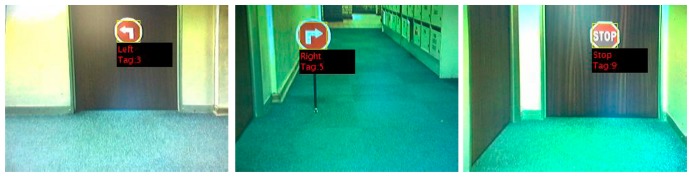
Left, right and stop signs recognised by the AGV and identification sign of each.

After a potential sign has been detected in consecutive video frames, the model identifies the sign to generate the appropriate command to be sent to the AGV. Examples of signs detected are shown in Figure 15.

### 4.2. Detecting the Colour for Different AGV Routes

Different methods for colour detection were investigated, which included:

- the RGB video signal implementing a tolerance for each colour signal representing this selected route colour;
- HSV signal rather than the RGB signal; and
- the YCbCr signal. This produced better results using the colour signals red (Cr) and blue (Cb).

**Figure 16.**
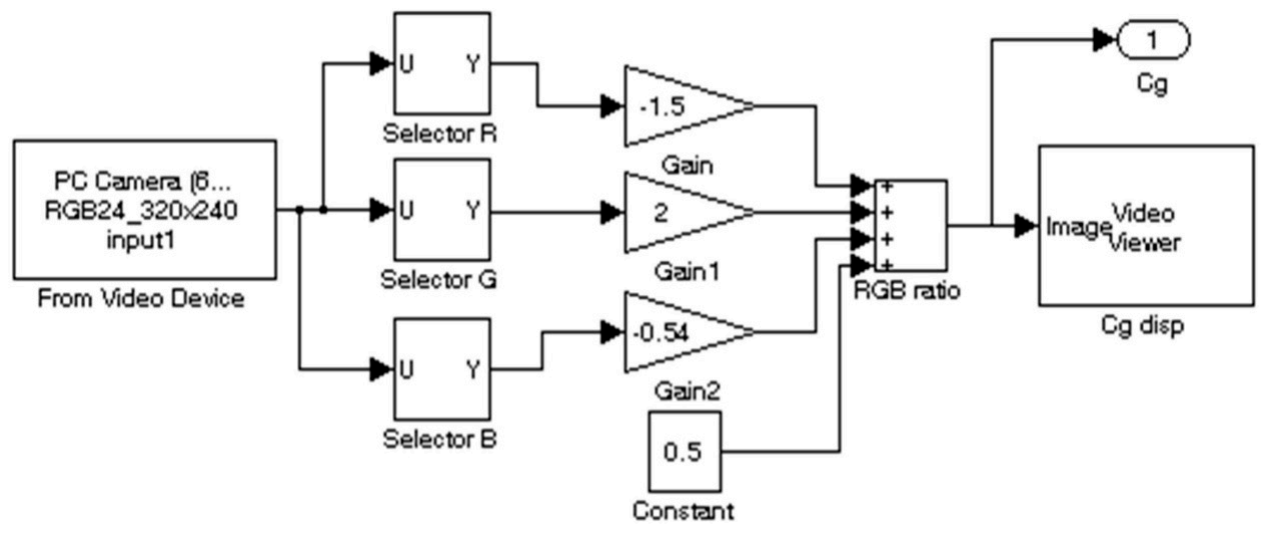
Matlab^®^ implementation of the Simulink^®^ model evaluated for a set Green signal.

Using Equations (2)–(4) below, and substituting typical constants for the *Y* signal, Equation (5) was derived. The equation was implemented with the Simulink^®^ model shown in Figure 16:

(2) $$Y=k_{r\cdot }R+k_{g\cdot }G+k_{b\cdot }B$$

(3) $$C_{B}=-k_{r\cdot }R-k_{g\cdot }G+k_{b\cdot }B$$

(4) $$C_{R}=+k_{r\cdot }R-k_{g\cdot }G-k_{b\cdot }B$$

(5) $$C_{g}=-1.5R+2G-0.54B+0.5$$

This method proved experimentally the most successful, as the colour selected made the output signal less sensitive to variations in different levels of lighting.

### 4.3. Implementing Sign Detection Command Control

Detecting the command signs successfully posed a problem with respect to the reaction time of the AGV to execute the relevant command. This made a difference in the distance from the sign to the specific position of the AGV.

The size of the different signs was standardised to be approximately 18 cm × 18 cm. By knowing the sign size, the distance from the AGV to the sign could be calculated using the number of pixels representing the image size recognised ([10], pp. 324–329). Table 3 gives a summary of the distance relevant to pixel count, obtained experimentally using the omnivision sensor.

A safe distance from the AGV to a sign or obstruction was found to be between 70 cm and 90 cm. This resulted in the choice of image size, representing the stochastic distance to a sign selected and evaluated, of approximately 84 × 84 pixels (total of 7056 pixels). The distance to the sign selection was developed to be a variable input in the Simulink^®^ model.

Determining this distance to the sign was achieved by using the area of the bounding box placed around a detected sign and then comparing this pixel count with the required size (total pixel count). When true, the relevant sign command detected was executed. Provision was made for a multiple count of signs detected in a single frame during consecutive frames.

**Figure 17.**
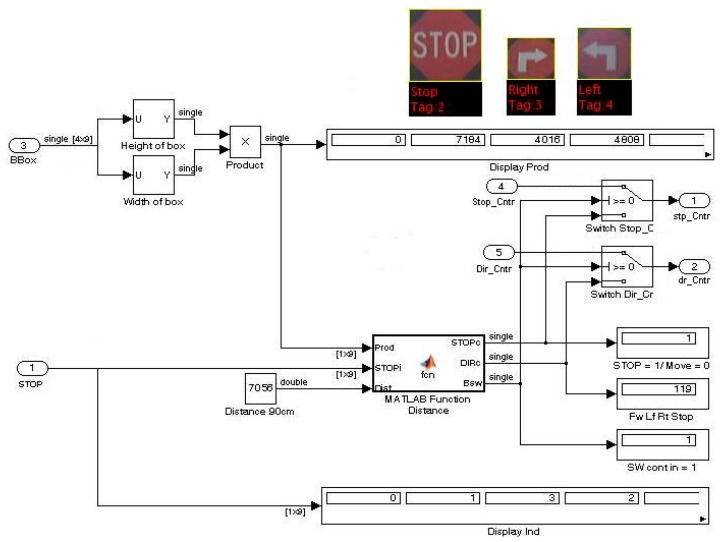
Simulink^®^ model implementing AGV motor control at a set distance depending on the area in terms of the number of pixels.

Figure 17 shows the implemented Simulink^®^ model block where the area of the detected sign (*Prod*) and the variable distance (*Dist*) is needed as input with the stop, direction and switch control signals generated as output.

**Figure 18.**
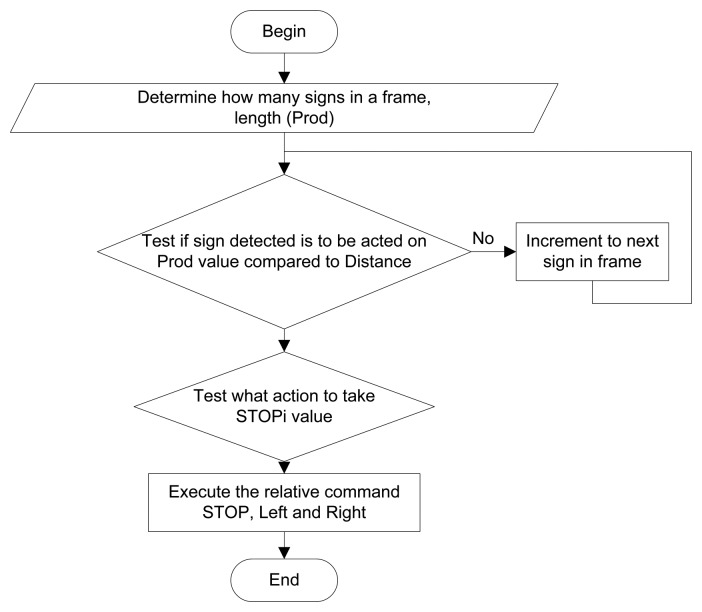
Flowchart for the *Distance* function block generating stop, direction and switch control.

**Figure 19.**
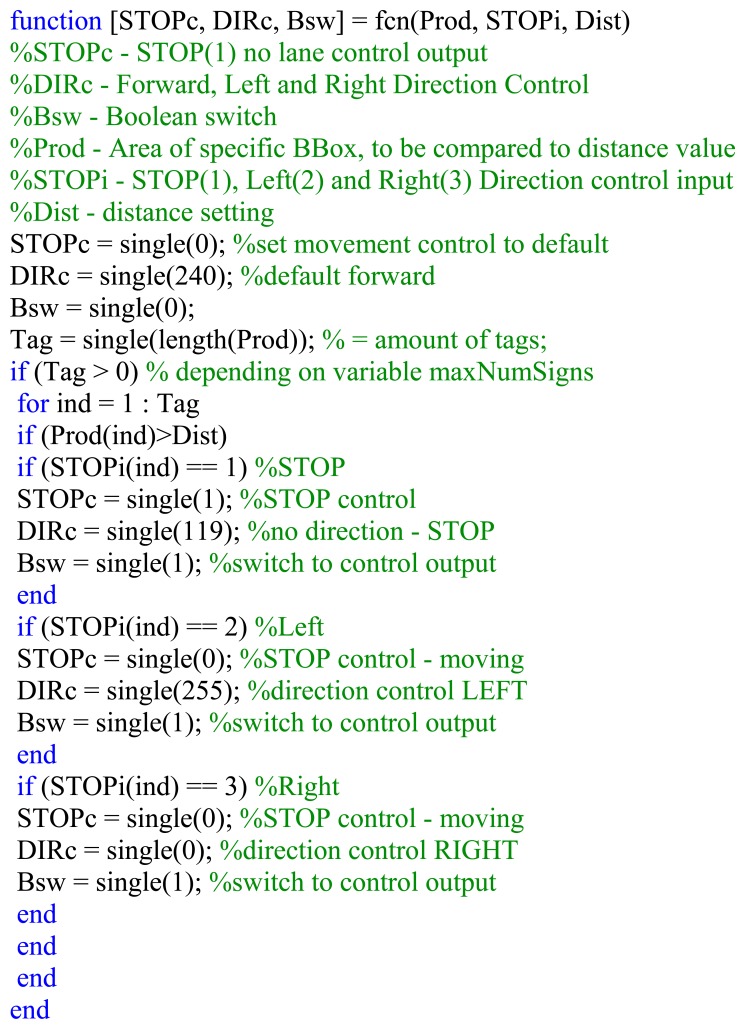
Abbreviated Matlab^®^ code for the *Distance* function block generating stop, direction and switch control.

Figure 18 shows the flowchart and Figure 19 indicates an abbreviated version of the Matlab^®^ function block code generating the control and switching signals. Only the forward, left, right and stop signals are shown for illustrative purposes.

## 5. Results and Discussion

**Figure 20.**
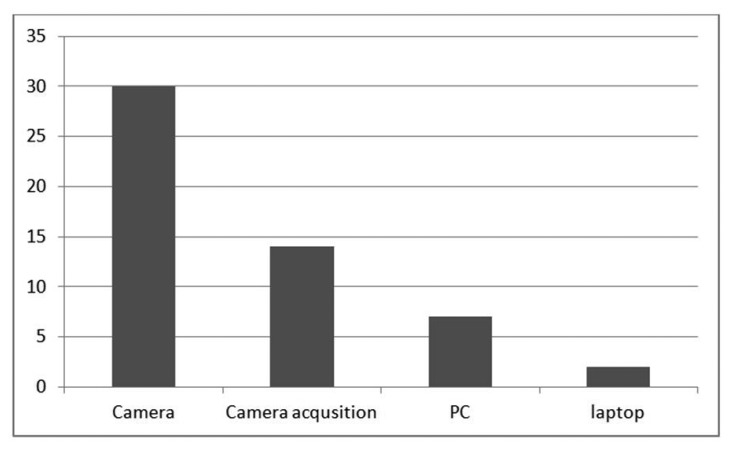
Decrease in frames per second along the image processing on the laptop platform relative to that of the PC.

Looking at the specifications of the PC and laptop used, the speed of the processor and size of RAM were the major factors that caused the difference in processing power. Figure 20 shows the drastic decrease in frames per second available to work with in image processing after each stage of the system, including that obtained by the PC for comparison. The frame rate of 30 frames per second available from the camera is decreased to almost 14 frames available after acquisitioning with a selected frame size of 96 × 128 pixels. This frame rate is further decreased to 2 frames per second after the omnivision conversion process available on the laptop and 7 frames per second on the PC for image processing.

### 5.1. AGV Performance as a Result of Using Matlab^®^

The maximum respective speeds of two different AGV types were 2.7 and 1.3 km per hour without using any vision—as depicted in Table 4. The maximum frame rates achieved by using the omnivision sensor in the study were 7 frames per second for the PC and 2 frames per second for the laptop, seen in Figure 2019. This information relates to a distance travelled of 36.5 cm per second with the slowest AGV, or approximately 18 cm travelled by the AGV per frame, using the laptop control which performs at 2 frames per second.

The speed of 18 cm per frame was clearly too fast to allow for image processing using the laptop. The AGV's speed needed to be reduced, because 6 to 8 frames per second were necessary for proper vision control. This meant that the AGV travelled more than a meter at 6 frames per second (18 cm × 6 frames = 108 cm). This is more than a typical turning circle distance (90 cm) allowed before a control decision could be made. Altering the speed to suit the processing time related to a speed of 6 cm per second, which was not suited for the final industry application. Thus the laptop processing speed was insufficient for such a vision sensor AGV control application.

### 5.2. Navigation and Control

In this section the performance of the route navigational system of the AGV was evaluated in terms of following the route as indicated by coloured signs and using vision [11]. As there was no provision made for localisation of the AGV by means of dead reckoning as in Swanepoel's ([12], pp. 41–44) research or using laser scanners and visual odometry as in Scaramuzza *et al.*'s work [13], the movement of the AGV needed to be monitored and noted by observation.

**Figure 21.**
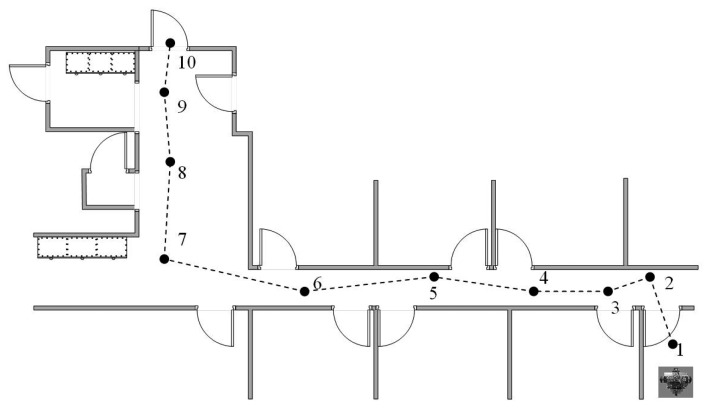
AGV position and orientation along a destined route plotted for evaluation.

The results were compared and noted with respect to the orientation of the AGV and its position on the route. What was evident was that the AGV could follow a set route with ease and that the commands generated from the navigation system did give the desired output to the AGV drive controls. Figure 21 shows typical plotted results and the position and orientation of the AGV for a specific evaluation performed.

**Figure 22.**
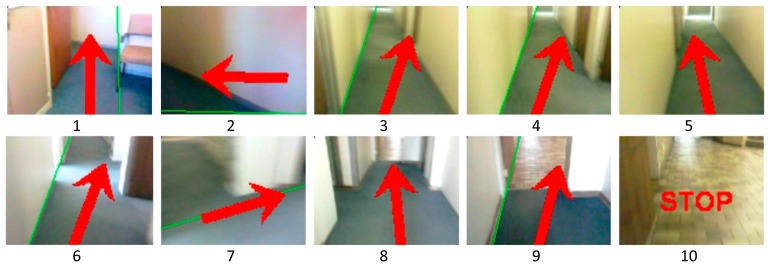
Corresponding frame captures for the positions indicated in Figure 21.

Figure 22 shows the corresponding direction control indication signals for monitoring purposes and AGV movement control.

### 5.3. Reconfigurable Ability of the Vision System

**Figure 23.**
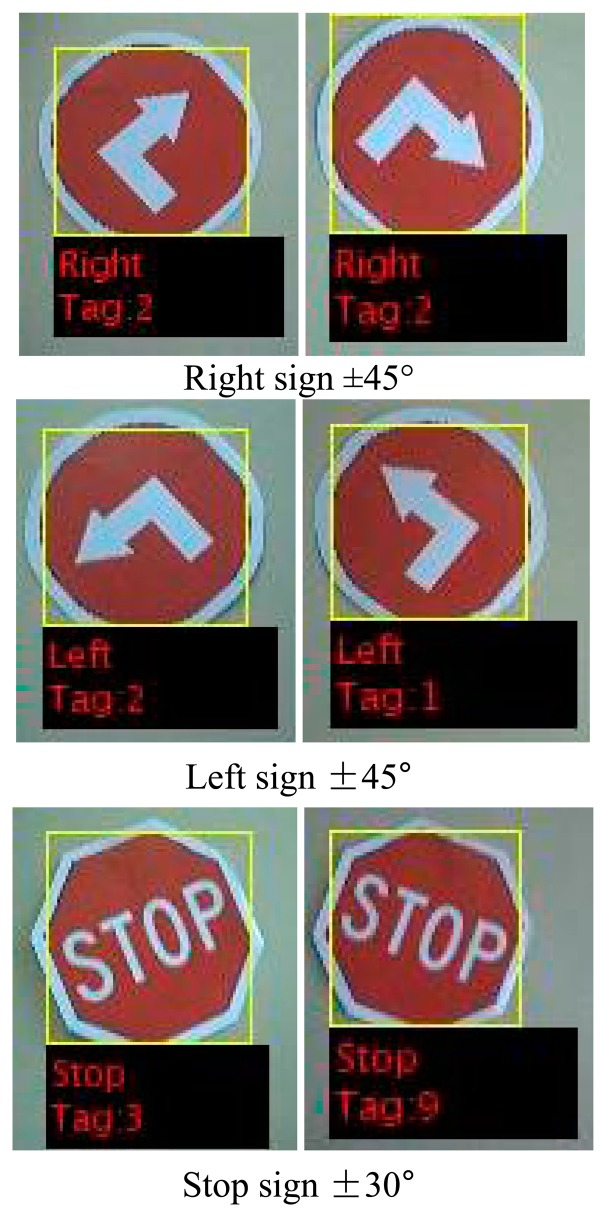
Indication of the degree at which the signs could be detected using sign recognition.

The sign recognition system provided the route reconfigurability to be applied by the operator by placing the applicable signs along the route for a specific AGV. The sign recognition system was designed and made provision for signs to be detected to a rotated angle of ±7.5°. The signs could however be detected to a maximum rotated angle of 45° for the left and right sign, and 30° rotated angle for the stop sign. Figure 23 indicates the results achieved in simulations, as it was never placed at this angle in the actual evaluation runs.

**Figure 24.**
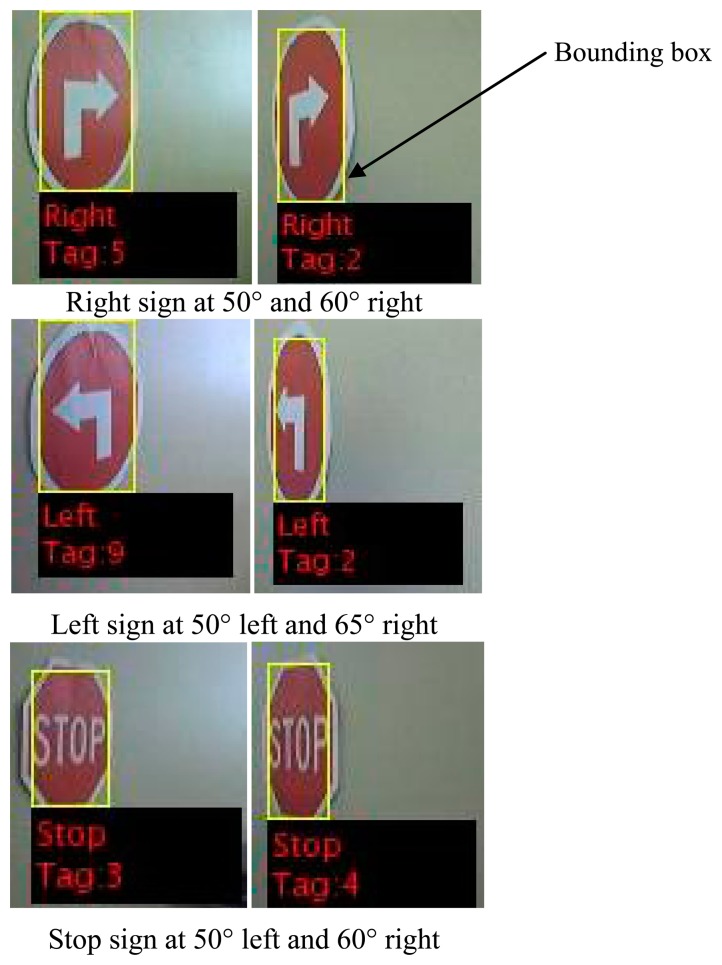
An indication of possible offset angles still resulting in successful sign recognition.

Encountering the signs at a horizontal offset angle also did not provide a problem, as the deviation from the straight-on position could vary by as much as 50° without causing a failure in recognising a sign, as can be seen in Figure 24.

**Figure 25.**
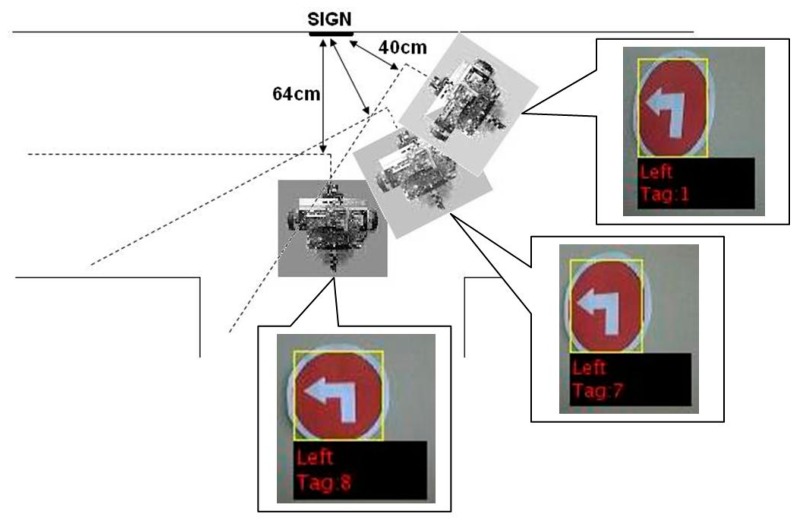
Distance from the sign determined by area at different angles of approach.

The AGV movement control acted on the signs control function at a predefined distance, set at 70 cm for evaluation purposes, determined by the set area of the bounding box. The distance between the sign and AGV was determined by the average area of the bounding box (seen in Figure 23) around the sign. The width of the bounding box depended most on the angle at which the AGV approached the sign, thus it had the biggest influence on the area of the bounding box, as the sign size was constant. The result was that, at a large horizontal angle deviation from head-on to the sign, the AGV acted on the sign command much later, resulting in a distance of reaction of between 64 cm and 40 cm. This did not pose any problems, as the size of the platform was relatively small. It is perhaps better illustrated in Figure 25.

## 6. Conclusions

The research covered in this article proved the viability of a developed omnidirectional conversion algorithm written in Matlab^®^. Selecting a webcam and making use of an area of interest enabled the saving of valuable computational time in converting an image.

Matlab^®^ was chosen as the complete software platform generating results, evaluating the camera setup and mirror configuration on a PC and later a laptop. The results obtained proved that the laptop processing time was too slow for omnivision purposes for the mobile system to be implemented in industry.

The navigational goals, using vision, as described in this article were successfully met by the developed AGV platform and the route navigation with the sign recognition and control implemented. A reconfigurable layout could be achieved with relative success using an AGV recognising only a set colour for its specific route.

## Figures and Tables

**Table 1.** Processor platform specifications.

**Personal Computer (PC)**	**Laptop**
Microsoft Windows XP	Microsoft Windows XP
Professional Version 2002 with Service Pack 3	Professional Version 2002 with Service Pack 3
Intel^®^ Core™ Duo CPU E8400 @ 3.00 GHz	Intel^®^ Core™ Duo CPU T7500 @ 2.20 GHz
2.98 GHz, 1.99 GB of RAM	789 Hz, 1.99 GB of RAM

**Table 2.** Frame rates incorporating different area of interest sizes compared to the results obtained on a PC and laptop.

**Input Frame Size**	**Output Frame Size**	**Frame Rate Obtained on PC**	**Frame Rate Obtained on Laptop**
640 × 480	720 × 186	3.5 frames per second	0.5 frames per second
640 × 480	720 × 186	4.5 frames per second	0.7 frames per second
640 × 480	360 × 186	7.5 frames per second	1.3 frames per second
640 × 480	180 × 96	14 frames per second	2.4 frames per second

**Table 3.** Summary of distance from AGV to signs with respect to image pixel count.

**Distance to a Sign**	**Approximate Pixel Count**
40 cm	174 × 174
50 cm	144 × 144
60 cm	120 × 120
70 cm	106 × 106
80 cm	96 × 96
90 cm	84 × 84
100 cm	76 × 76
110 cm	66 × 66

**Table 4.** Comparative speeds of the 3- and 4-wheeled NI AGV's used in the research without vision.

	**3 Wheel AGV**	**4 Wheel AGV**
Maximum speed obtained in forward/reverse without vision	2.7 km/h	1.3 km/h
Individual speeds denoted in meter per minute	45.24 m/min	21.9 m/min
Individual speeds denoted in centimetre per second	75.4 cm/s	36.5 cm/s
